# Risk of cervical intraepithelial neoplasia grade 2 or worse in women aged ≥ 69 referred to colposcopy due to an HPV-positive screening test

**DOI:** 10.1186/s12885-023-10888-1

**Published:** 2023-05-05

**Authors:** Berit B. Booth, Mette Tranberg, Line W. Gustafson, Anne G. Christiansen, Helle Lapirtis, Lisa M. Krogh, Ina Marie D. Hjorth, Anne Hammer

**Affiliations:** 1 Department of Obstetrics and Gynecology, NIDO - Centre for Research and Education, Gødstrup Hospital, Hospitalsparken 15, Herning, 7400 Denmark; 2 University Research Clinic for Cancer Screening, Department of Public Health Programmes, Randers Regional Hospital, Randers, Denmark; 3 Department of Clinical Medicine, Aarhus University, Aarhus, Denmark; 4 Department of Obstetrics and Gynecology, Randers Regional Hospital, Randers, Denmark; 5 Department of Obstetrics and Gynecology, Viborg Regional Hospital, Viborg, Denmark; 6 Department of Obstetrics and Gynecology, Horsens Regional Hospital, Horsens, Denmark

**Keywords:** Cervical cancer screening, Human papillomavirus, Colposcopy, Cervical intraepithelial neoplasia, Loop electrosurgical excision procedure, Postmenopausal women

## Abstract

**Background:**

Cervical cancer incidence and mortality rates are high in older women in many developed countries, including Denmark. Therefore, Danish women aged 69 and older were invited for one additional human papilloma virus (HPV) based screening test in 2017. Here, we describe the clinical management and detection rate of cervical intraepithelial neoplasia grade 2 or worse (CIN2 +) in screen-positive women referred for colposcopy.

**Methods:**

We conducted an observational study in public gynecology departments in Central Denmark Region, Denmark. Women were eligible for enrolment if they were aged 69 + in 2017, HPV positive on a screening test taken between April 20^th^, 2017, and December 31^st^, 2017, and had been referred for direct colposcopy. Data on participants’ characteristics, colposcopic findings, and histological outcomes were collected from medical records and the Danish Pathology Databank. We estimated the proportion of women with CIN2 + at the first colposcopy visit and at end of follow up including 95% confidence intervals (CIs).

**Results:**

A total of 191 women were included with a median age of 74 years (IQR: 71—78). Most women (74.9%) did not have a fully visible transformation zone at colposcopy. At the first visit 170 women (89.0%) had a histological sample collected, 34 of whom (20.0%, 95% CI 14.3–26.8%) had CIN2 + diagnosed, 19 had CIN3 + , and two had cervical cancer). During follow-up additional CIN2 + were detected resulting in a total of 42 women (24.4%, 95% CI: 18.2–31.5%) being diagnosed with CIN2 + , 25 with CIN3 + , and three with cervical cancer. When restricting to women with paired histologic results (i.e., biopsies and a loop electrosurgical excision procedure (LEEP) specimen), we found that CIN2 + was missed in 17.9% (95% CI 8.9–30.4%) of biopsies compared to the LEEP.

**Conclusion:**

Our findings suggest a potential risk of underdiagnosis in older postmenopausal women referred to colposcopy**.** Future studies should explore potential risk-markers for discrimination of women at increased risk of CIN2 + from those at low risk, as this would reduce risk of underdiagnosis and overtreatment.

**Supplementary Information:**

The online version contains supplementary material available at 10.1186/s12885-023-10888-1.

## Introduction

Cervical cancer incidence rates are higher in older women compared to younger women, and older women are more likely to be diagnosed with late-stage cervical cancer and die from the disease [1, 2]. Previous studies suggest that this may be because older women may not have had the opportunity to be screened and that cytology-based screening may fail to detect precancer and cancer in older women due to low sensitivity [3, 4]. These findings may suggest a need to continue screening beyond the age of 65 using a different screening method such as HPV-based screening. Several studies have clearly demonstrated that HPV-based screening is superior to cytology-based screening, also in older women [5–7]. However, diagnostic work-up of older women who screen positive is challenging, particularly because of atrophy and the retraction of the transformation zone, which makes complete visualization of the transformation zone (TZ) and potential lesions impossible. This may result in an increased risk of missing disease by up to twofold [8], potentially resulting in a delay in diagnosis [9]. To improve the detection and reduce risk of missing disease in this group of women, endocervical curettage (ECC) can be performed but remains controversial [10]. Although ECC may improve the detection rate of cervical precancer [11], not much is known about the diagnostic accuracy of ECC in older postmenopausal women. To ensure accurate diagnostics and treatment in women with incomplete visualization of the TZ some guidelines recommend that a diagnostic loop electrosurgical excision procedure (LEEP) be considered [12–14]. However, these recommendations are based on expert opinion as there is limited empirical evidence available on how to clinically manage older women.

In Denmark, women aged 69 + (i.e., women born before 1948) were invited for an additional HPV-based screening test in 2017 [15] as an initiative to improve cervical cancer prevention among older birth cohorts with insufficient screening history [4]. Here, we aimed to describe clinical management and the proportion of women with CIN grade 2 or worse (CIN2 +) in women aged 69 + referred to colposcopy.

## Materials and methods

We conducted an observational study from January to December 2020 in Central Denmark Region, which compromises approximately 20% of the Danish population. In Denmark, cervical cancer screening, including follow-up, diagnostic work-up and treatment, is free of charge. Women aged 23–64 are invited for regular screening every three to five years depending on age and screening method. From April 17 through December 31, 2017, women born before 1948 (i.e., aged 69 + at that time) were invited for one additional HPV screening test [4, 15]. HPV DNA testing was performed using the clinically validated COBAS 4800 assay (Roche Diagnostics), which allows for individual detection of HPV16 and HPV18, and pooled detection of 12 other high-risk HPV types (31,33,35,39,45,51,52,55,56,58,66,68). Women were referred directly for colposcopy if they were positive for HPV16 and/or HPV18, or if they were positive for other HPV types than HPV16/18 and had atypical squamous cells of undetermined significance or worse (ASC-US +) on reflex cytology. Women positive for HPV types other than HPV16/18 with normal cytology were recommended repeat HPV testing after one year and were only referred for colposcopy if HPV positive. Cytology was classified according to the 2014 Bethesda classification. As reported elsewhere, a total of 75,937 women were invited for screening in Central Denmark Region, 31.2% of whom participated, and the HPV prevalence was 4.8% in this population [16, 17] (Additional file 1).

Diagnostic work-up including colposcopies were performed in private gynecology clinics or public hospitals. During the study period there was no national clinical guidelines on how to clinically manage and treat HPV-positive women outside the typical screening age (i.e., age 65 and above). National clinical guidelines recommend that women of screening age should have biopsies collected regardless of the presence of visible lesions, with surgical treatment being recommended in the case of CIN2 + in women outside of reproductive age [18]. Of note, collection of endocervical cytology or ECC is not routinely recommended in the Danish guidelines but may be performed depending on the colposcopist’s preference. Of note, in this paper, ECC was mainly performed at one gynecological department.

Using electronic medical records, we identified women who were referred for colposcopy to a gynecology department at any public hospital in Central Denmark Region (i.e., Herning, Randers, Viborg, and Horsens) due to an HPV-positive screening test in the study period from April 20^th^ 2017 to December 31^st^ 2017. Eligible women had 1) a liquid-based cytology sample collected for HPV testing as part of the national one-time screening offer in the study period [15], and 2) were born before 1948. Women were excluded if their cervical sample was collected as part of follow-up for previous dysplasia, had undergone hysterectomy, or if they were examined in a private gynecology clinic as we had no access to medical records on these women.

From electronic medical records we collected information on colposcopic findings at the first visit including type of transformation zone (i.e., TZ1, TZ2, TZ3 as defined according to the 2011 International Federation of Cervical Pathology and Colposcopy nomenclature [19]). Information on previous screening history (i.e., at least one record of screening vs no record of screening), and results of subsequent cervical biopsies, cervical smears, ECC, and LEEP were collected from the national Danish Pathology Databank [20]. The Danish Pathology Databank is a complete database that includes all pathology examinations dating back to 1997, and for some examinations back to 1970 [20]. Data was collected at an individual level using the central personal registration number (CPR), which is a unique code assigned to residents at birth or upon immigration [21]. Women were followed-up until December 31, 2020.

Histological diagnoses were graded according to the CIN classification system [22]; normal (including inflammation and non-specific reactive features), ungradable CIN (i.e., the full height of the epithelium is not discernible), CIN1, CIN2, CIN3, AIS, or cancer. If more than one histological diagnosis was recorded for the woman in the time span between the date of the HPV-positive screening test and end of follow-up (December 2020), the most severe diagnosis was used. Histological diagnoses were grouped into ≤ CIN1 (normal and CIN1) and CIN2 + (CIN2 and above), including ungradable CIN as these cases are clinically managed as CIN2 + . In case multiple HPV types were detected, we applied a hierarchical classification, assuming HPV 16 and/or 18 were the causal genotypes [23]. If both colposcopy-directed biopsies and random biopsies were taken, these cases were recorded as having directed biopsies taken. If it was not specified whether biopsies were directed or random, biopsies were recorded as random.

Our primary outcome was CIN2 + as this is the threshold for treatment in older women. Secondary outcomes were CIN3 + and cancer. We reported the overall number and proportion of CIN2 + , CIN3 + , and cancer after the first visit and at the end of follow-up, including corresponding 95% confidence intervals (95% CI). In a sub-analysis, we compared the CIN2 + detection in biopsies/ECC vs LEEP in women with paired histologic results. Data was entered and stored in REDCap [24]. Stata 16.1 analytic software (Stata Corp LP, College Station, TX) was used for data analysis.

## Results

**Fig. 1:**
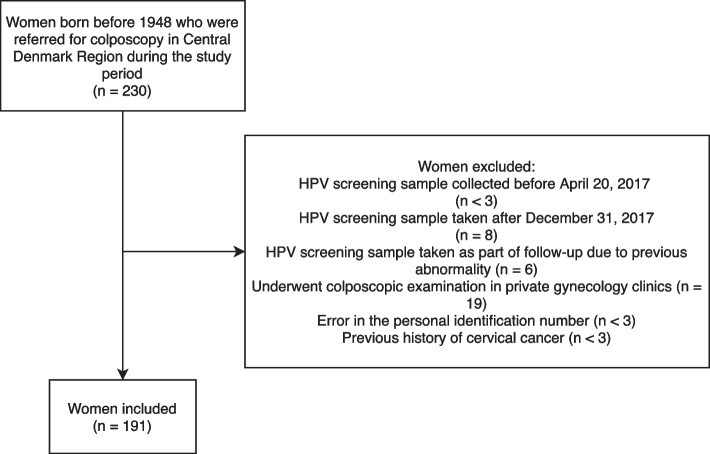
Flow diagram of women included in the present study

We identified 230 women born before 1948 who had a screening sample collected in the study period. Of these, 191 women (83.0%) met the inclusion criteria and were included (Fig. 1). The median age of included women was 74 years (IQR: 71—78) (Table 1). HPV 16 was the most common genotype (55.0%), followed by other high-risk types than HPV16/18 (29.8%), and HPV18 (15.2%) (Table 1). Most women (83.8%) had HPV 16, 18, or other HPV types only, while 16.2% had a mixture of HPV 16, 18 and/or other HPV types detected (data not shown). Most women (98.4%) had at least one record of a previous cervical cytology sample in the Danish Pathology Databank prior to 2017, 18.9% (*n* = 36) had a previous record of ASCUS + and/or CIN1 + , and 6.8% (*n* = 13) had a previous record of a LEEP (Table 1).

**Table 1:** Basic characteristics of women born before 1948 who were referred for colposcopy due to an HPV-positive screening test (*n* = 191)

	**n (%)**
**Median age in years (IQR)**	74 (71–78)
**HPV type in referral screening test**^a^	
HPV 16	105 (55.0%)
HPV 18	29 (15.2%)
Other high-risk HPV	57 (29.8%)
**Cytology triage in baseline referral screening test in women with other high-risk HPV**	
Normal	6 (10.5%)
ASCUS	13 (22.8%)
LSIL	11 (19.3%)
ASC-H	12 (21.1%)
HSIL	15 (26.3%)
**Number of colposcopy visits**	
1	134 (70.2%)
2	36 (18.8%)
3	16 (8.4%)
4	5 (2.6%)
**At least one previous record of a cervical smear**^b^	
Yes	188 (98.4%)
No	3 (1.6%)
**Previous record of ASCUS + or CIN1 + **^b^	
Yes	36 (18.9%)
No	155 (81.2%)
**Previous record of a LEEP**^b^	
Yes	13 (6.8%)
No	178 (93.2%)

Of note, percentages may add up to more than 100% due to rounding

*Abbreviations: IQR* Inter quartile range, *ASCUS* Atypical Squamous Cells of Undetermined Significance, *LSIL* Low-​grade Squamous Intraepithelial Lesions, *ASC-H* Atypical squamous cells, cannot exclude High-grade, *HSIL* High grade squamous intraepithelial lesion, *LEEP* Loop Electrosurgical Excision Procedure

^a^ Hierarchical classification of HPV genotypes

^b^ Previous record in the Danish Pathology Databank

Among 191 HPV-positive women referred to colposcopy, most women had a TZ3 (*n* = 143, 74.9%) at the first colposcopy visit (Table 2). In 171 (89.5%) of these women no colposcopic abnormalities were reported (Table 2), and most women had random biopsies (75.9%) collected. Reasons for abstaining from collection of biopsies at the first visit were mainly cervical stenosis, no visible lesions, or patient wishes (data not shown). A total of 170 women (89.0%) had histological samples collected (i.e., biopsies, ECC, or LEEP) at or immediately after the first colposcopy visit, with most women having biopsies collected (94.7%, 161/170). In these biopsies the TZ was not represented in 65.8% (106/161) of women, while 32.9% (53/161) of women had the TZ represented in at least one biopsy, and in 1.2% (2/161) of women it was not reported. Of women who had a histological sample collected, 34 (20.0%, 95% CI 14.3–26.8%) women had CIN2 + diagnosed, 19 (11.2%, 95% CI 6.9 – 16.9) had CIN3 + , and two (1.2%, 95% CI 0.1 – 4.2%) were diagnosed with cervical cancer (Table 2).

**Table 2:** Colposcopic findings at the first colposcopy visit among HPV screening-positive women, overall and stratified by histology

			**Histological sample collected**^c^ **(*n* = 170) (%)**			
	**All women** **(*n* = 191, 100%)**		** ≤ CIN1** **(*n* = 136, 80%)**		**CIN2 + ** **(*n* = 34, 20%)**	
	**n (%)**	**(95% CI)**	**n (%)**	**(95% CI)**	**n (%)**	**(95% CI)**
**Transformation zone type recorded**						
TZ1	15 (7.9%)	4.5–12.6%	7 (5.2%)	2.1–10.3%	8 (23.5%)	10.8–41.2%
TZ2	16 (8.4%)	4.9–13.2%	15 (11.0%)	6.3–17.5%	< 3	-
TZ3	143 (74.9%)	68.1–80.9%	101 (74.3%)	66.1–81.4%	22 (64.7%)	46.5–80.3%
Not reported	17 (8.9%)	5.3–13.9%	13 (9.6%)	5.2–15.8%	3 (8.8%)	1.9–23.7%
**Colposcopic abnormality**^a^						
Acetowhite	10 (5.2%)	2.5–9.4%	6 (4.4%)	1.6–9.4%	4 (11.8%)	3.3–27.5%
Punctuations or mosaic vessel patterns	5 (2.6%)	0.9–6.0%	2 (1.5%)	0.2–5.2%	3 (8.8%)	1.9–23.7%
Atypical vessels	11 (5.6%)	2.9–10.1%	8 (5.9%)	2.6–11.3%	3 (8.8%)	1.9–23.7%
Discoloration	< 3		< 3		0	
No visible lesions	171 (89.5%)	84.3–93.5%	123 (90.4%)	84.2–94.8%	27 (79.4%)	62.1–91.3%
**Median number of biopsies collected (range)**	3 (0–7)		3 (1–7)		4 (1–7)	
**Type of histological sample**^b^						
Colposcopy-directed biopsies	17 (8.9%)	5.3–13.9%	12 (8.8%)	4.6–14-9%	5 (14.7%)	5.0–31.1%
Random biopsies	145 (75.9%)	69.2–81-8%	116 (84.6%)	78.2–90.8%	28 (82.4%)	65.5–93.2%
Endocervical curettage	29 (15.2%)	10.4–21.1%	23 (16.9%)	11.0–24.3%	5 (14.7%)	5.0–31.1%
Diagnostic LEEP	17 (8.9%)	5.3–13.9%	10 (7.3%)	3.5–13.1%	7 (20.6%)	8.7–37.9%
LEEP due to abnormal histological sample	22 (11.5%)	7.3–16.9%	0	-	22 (64.7%)	46.5–80.3%

Of note, percentages may add up to more than 100% due to rounding

^a^ Adds to more than 100% as some women had more than one abnormality detected at colposcopy

^b^ If both colposcopy-directed biopsies and random biopsies were taken, these cases were recorded as having directed biopsies taken. If it was not specified whether biopsies were directed or random, these cases were recorded as random

^c^ Based on worst histopathological examination of cervical biopsies, ECC and/or LEEP

Compared to women diagnosed with CIN1 at the first visit, women diagnosed with CIN2 + were significantly more likely to have a TZ1 (23.5% vs 5.2%), have any abnormality detected (20.6% vs 9.6%), and have undergone a diagnostic LEEP (20.6% vs. 7.3%) (Table 2). We found no difference between the two groups with respect to ECC.

With respect to data from the entire study period, a total of 172 (90.1%) had one or more histological samples collected (i.e., biopsy, ECC or LEEP). Of these, 42 women (24.4%, 95% CI: 18.2–31.5%) had CIN2 + detected, 25 (14.5%, 95% CI 9.6 – 20.7) had CIN3 + detected, and three (1.7%, 95% CI 0.4 – 5.0) were diagnosed with cervical cancer.

LEEP was performed in 62 women (32.4%) at some point during the study, with 56 (29.3%) having both cervical biopsies and/or ECC and a LEEP performed. Despite having no evidence of CIN2 + in the biopsies or the ECC, 10 women had CIN2 + (10/56, 17.9%, 95% CI 8.9–30.4%) detected in the LEEP specimen (data not shown). When restricting to women with biopsies and LEEP, similar findings were found.

## Discussion

In this group of older HPV-positive women referred for colposcopy, 20.0% had CIN2 + detected at the first visit and 24.4% had CIN2 + detected at some point during the study period. Most women diagnosed with CIN2 + had no abnormalities visualized at colposcopy. Cervical biopsies/ECC underestimated the detection of CIN2 + by 17.9% compared with the LEEP result. As 73.3% of women only had cervical biopsies or an ECC collected during the study period, despite having a TZ3 at colposcopy, the proportion of CIN2 + reported in this study is likely underestimated. Further, the majority of biopsies taken (65.8%) did not include the TZ, likely contributing further to the underestimation of high-grade disease.

The proportion of women with CIN2 + in our study (20.0% and 24.4%) was comparable to that reported in a previous Swedish study (23%) on older women with a persistent HPV infection but was higher than the one reported in a previous population-based Danish study (18%) [17, 25, 26]. Given differences in screening and referral strategy, age of the population, proportion of women with a TZ3, and clinical management, it is challenging to make any formal comparison across studies.

According to a Swedish study collection of random biopsies and ECC were useful clinical tools in detecting CIN2 + in older women with TZ3 in the absence of visible lesions [25]. Our results, however, indicate that we should be cautious when relying only on blind random biopsies, even when a median of 3 biopsies were taken. We found that biopsies underestimated CIN2 + in 17.9% of cases compared with the LEEP diagnosis, which is similar to Gustafson et al. who showed a 17.7% difference in older women who all underwent both biopsy and LEEP [8]. These findings suggest that women with TZ3 are at increased risk of having disease missed and that collection of blind biopsies may result in an insufficient diagnostic work-up because the lesion may be located high in the cervical canal. Therefore, colposcopists should be cautious when relying solely on the result of cervical biopsies in this group of women as a negative biopsy result does not necessarily infer no cervical disease is present. Given that national estimations of CIN2 + and CIN3 + detection rates in older women may be based on the results of cervical biopsies, immediate risk-assessment may be biased as well.

According to the British, Swedish, Australian, and American guidelines, a diagnostic LEEP may be considered when the squamocolumnar junction is not fully visualized [12–14, 27]. However, performing a LEEP in all HPV-positive older women will likely lead to overtreatment and potential surgical complications, such as stenosis, which may compromise subsequent follow-up. HPV may also still be present after a LEEP as it has been shown that HPV clearance may be delayed after LEEP, especially in older women [28]. Balancing the harms of potential overtreatment versus underdiagnosis is of great importance in clinical management of older HPV-screen-positive women referred for colposcopy. Referral for colposcopy is a known harm of screening as it can lead to psychological distress and anxiety [29–31]. However, a previous study has found lower anxiety levels in older women undergoing colposcopy and treatment (median age 67.7 years) compared to women aged 23–50 [32, 33]. Additionally, a recent Danish qualitative study demonstrated that older post-menopausal women preferred diagnostic LEEP over continued surveillance, even if this would result in increased risk of overtreatment [34]. From a clinical perspective, a shared decision-making tool could be helpful for women and their health-care provider as this may enable a better discussion of pros and cons, particularly because the current literature on this subject is limited.

The subject of when women should exit the cervical cancer screening program has gained much attention lately [35], particularly due to decreasing hysterectomy rates and an increasing life expectancy, leaving more women at-risk. However, as the preventive effect of cervical cancer screening not only relies on the uptake and effectiveness of the screening test but also on accurate diagnostics of those who screen positive, it is critical that clinicians ensure accurate diagnostics and adequate treatment of screen-positive women with minimal harm if screening is to be performed in this population. In our study it was clear that women were followed differently depending on hospital traditions and colposcopists’ experience. For example, ECC was only performed in 16.8% of women, and these were mainly performed in one hospital.

Further research is needed to correctly identify the HPV-positive older women who are at greatest risk of CIN2 + . Cytology triage may not be the best solution in older HPV-positive women as cytology has been shown to have a lower sensitivity in this population [36]. Furthermore, triage by partial HPV genotyping (i.e., HPV 16/18) may also be suboptimal as the relative contribution of HPV16/18 in cervical cancer declines with increasing age, reaching about 45% in women aged 70 + . [23] Biomarkers like p16/Ki67 dual stain-cytology or DNA methylation of certain host genes show promising results in triage of younger women who screen HPV positive [37, 38]. Nevertheless, these results may not be generalizable to an older population of women, where collection of cervical samples is challenging. However, a recent Danish study on screen-positive postmenopausal women suggested that p16/Ki67 dual stain-cytology can be a useful tool to determine which women can safely undergo repeated cervical sampling rather than a diagnostic LEEP [39]. This could potentially minimize the risk of overtreatment while reducing risk of underdiagnosis. Before better triage methods are widely available, clear consensus guidelines on how to best manage and follow-up these older women are critically needed.

### Strengths and limitations

Strengths of this study were the use of individual-level data from electronic medical records. Further, we used the national Danish Pathology Databank, which ensured a complete record of previous cervical cancer screening history and valid results on histopathologic outcomes at an individual level. Limitations of the study were that those women with HPV16 or 18 were referred directly for colposcopy and women with non-HPV16/18 genotypes were only referred directly for colposcopy if cytology was abnormal (ASC-US +). Therefore, we were unable to include women with non-HPV 16/18 and normal cytology. With this selection bias it appears that HPV16 was the most common HPV type in these women despite that a previous study reported non-HPV16/18 genotypes to be more common in older women compared to HPV16 and 18 [40]. This may have underestimated the true prevalence of non-HPV 16/18, and may have affected the generalizability of our data to other populations. One could anticipate that this selection could also have underestimated the detection rate of CIN2 + . However, this limitation is considered less likely as this research group previously found that older women found to be HPV positive were more likely to have CIN2 + present in LEEP if cytology was abnormal than if cytology had been normal at referral [8]. Due to the lack of clinical guidelines, women were managed differently between the hospitals and within each hospital dependent on the colposcopist. This makes it difficult to compare data but reflects the everyday clinical challenges colposcopists are facing. Finally, the small sample size makes the results less robust.

## Conclusion

We found that one in four women aged 69 + referred for colposcopy due to an HPV-positive screening test were diagnosed with CIN2 + . However, the overall proportion of CIN2 + may be underestimated as 73.3% of women with TZ3 had insufficient diagnostic work-up. Future studies should explore the use of biomarkers for identification of screen-positive postmenopausal women who at the first colposcopy visit could be at increased risk for CIN2 + , thereby reducing risk of overtreatment and simultaneously secure adequate diagnostics. In the meantime, shared decision making between the clinician and woman discussing the pros and cons of repeated cervical follow-up versus diagnostic LEEP will be essential.

## Supplementary Information

**Additional file 1.** Flow diagram of Women aged 69+ (i.e., women born before 1948) invited for an additional HPV-based screening test in Central Denmark Region.

## Data Availability

The data that support the findings of this study are available from each hospital administration, but restrictions apply to the availability of these data, which were used under license for the current study, and so are not publicly available. Data are however available from the authors upon reasonable request and with permission of each hospital administration.

## Abbreviations

95% CI: 95% Confidence interval
CIN: Cervical Intraepithelial Neoplasia
ECC: Endocervical Curettage
HPV: Human papillomavirus
IQR: Interquartile range
LEEP: Loop Electrosurgical Excision Procedure
TZ: Transformation Zone
