# Supplementary material for: Risk of cervical intraepithelial neoplasia grade 2 or worse in women aged ≥ 69 referred to colposcopy due to an HPV-positive screening test
Source: BMC Cancer. 2023 May 5;23:405. doi: 10.1186/s12885-023-10888-1 (PMC10161414; doi:10.1186/s12885-023-10888-1)
Supplement: Supplementary file 1 — Additional file 1. Flow diagram of Women aged 69+ (i.e., women born before 1948) invited for an additional HPV-based screening test in Central Denmark Region. [file 12885_2023_10888_MOESM1_ESM.docx]

**Additional file**

**Additional file 1: Flow diagram of Women aged 69+ (i.e., women born before 1948) invited for an additional HPV-based screening test in Central Denmark Region**

**
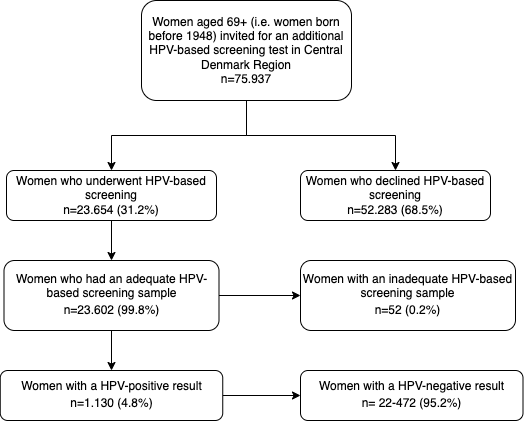
**

*Reference no. 16: The Danish Quality Database for cervical cancer screening (Dansk Kvalitetsdatabase for Livmoderhalskræftscreening – DKLS) “Resultater fra Engangstilbuddet: Livmoderhalskræftscreening blandt danske kvinder født før 1948” 1. delrapport. 2019
